# Supplementary material for: Interplay between ferroelectricity and metallicity in BaTiO3
Source: J Mater Chem C Mater. 2021 Jun 22;9(27):8640–9. doi: 10.1039/d1tc01868j (PMC8280966; doi:10.1039/d1tc01868j)
Supplement: TC-009-D1TC01868J-s001 [file TC-009-D1TC01868J-s001.pdf]

Electronic Supplementary Material (ESI) for Journal of Materials Chemistry C.
This journal is © The Royal Society of Chemistry 2021

Interplay between Ferroelectricity and Metallicity in $BaTiO_3$

Veronica F. Michel, Tobias Esswein,* and Nicola A. Spaldin

Materials Theory, Department of Materials, ETH Zürich, Wolfgang-Pauli-Strasse 27, 8093 Zürich, Switzerland

*Corresponding author: *tobias.esswein@mat.ethz.ch*

Electronic Supplementary Information

If not otherwise specified in the caption of the figures, the computational parameters used correspond to the ones described in the methods section of the main text.

1 Undoped Tetragonal $BaTiO_3$

Berry-phase Calculation

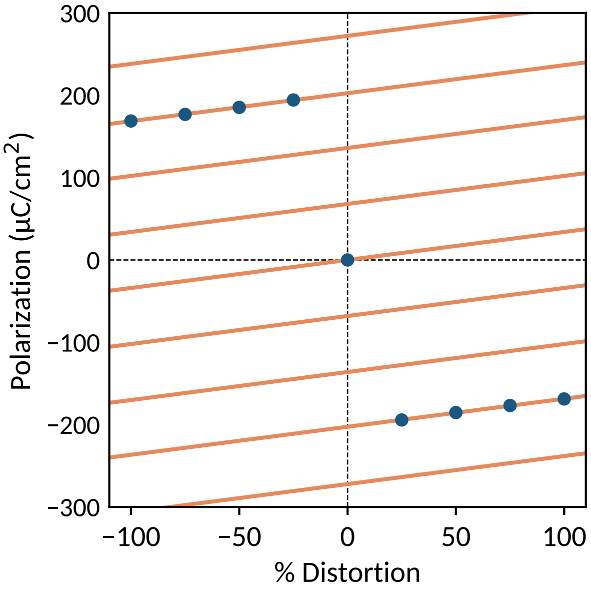

Figure 1: Berry-phase calculation for tetragonal $BaTiO_3$. The calculated spontaneous polarization of the system is 34 $\mu C/cm^2$.

2 Background-charge Doped Pseudocubic $BaTiO_3$ Unit Cell

Density of States

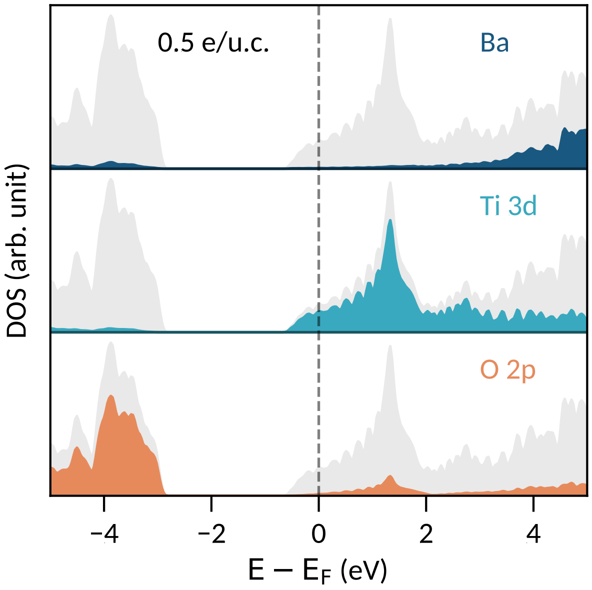

Figure 2: Density of states for pseudocubic $BaTiO_3$ doped with 0.5 e/u.c.

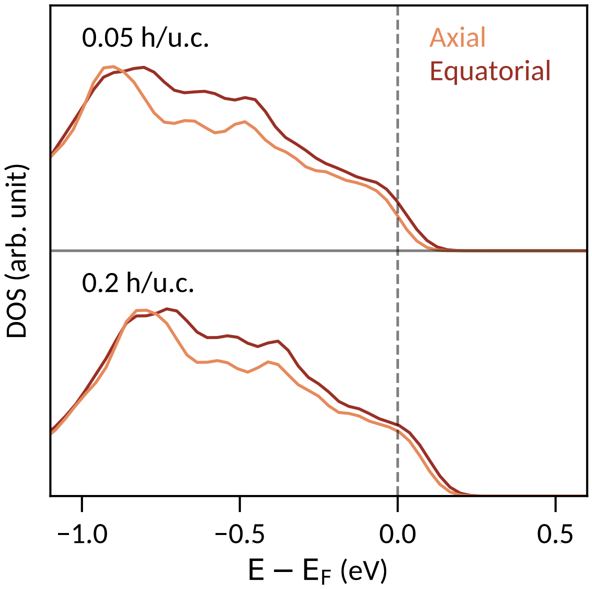

Figure 3: Oxygen site-resolved densities of states for pseudocubic $BaTiO_3$ doped with 0.05 h/u.c. (top) and 0.2 h/u.c. (bottom).

3 Impurity-doped $BaTiO_3$ Supercells

Polarization, Volume and Tetragonality

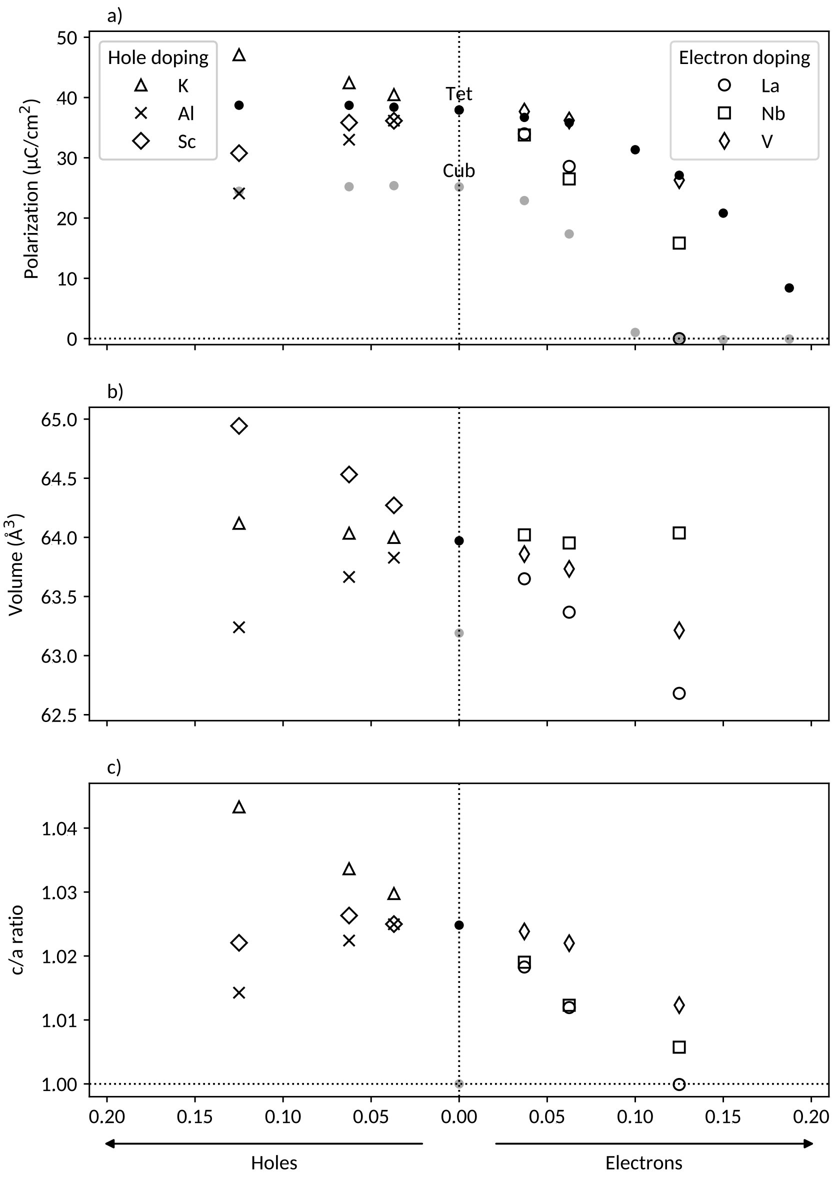

Figure 4: Polarization (a), average volume (b) and c/a ratio (c) of doped $BaTiO_3$ as a function of the charge-carrier concentration (electrons on the right, holes on the left). The charge carriers are introduced through doping with impurity atoms in 2×2×2, $2\sqrt{2}\times2\sqrt{2}\times2$ and 3×3×3 supercells and their charge-carrier concentrations are 0.125, and 0.0625 and 0.037 carriers/u.c., respectively. The k-point grids used for these calculations are 12×12×12, 6×6×6 and 4×4×4, respectively. The systems are labeled with the name of their respective dopant. The black and grey dots correspond to the tetragonal and pseudocubic background-charge references.

Bond Ratios

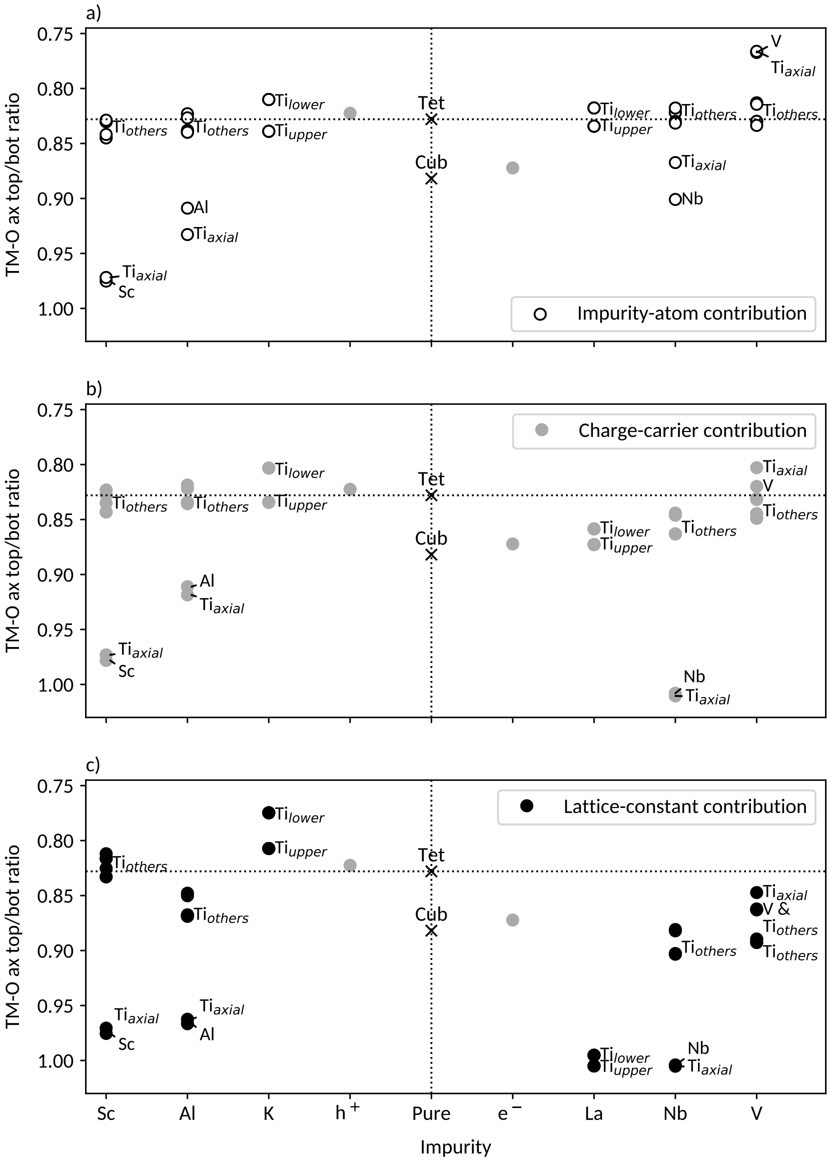

Figure 5: Bond ratios of the transition metal - oxygen axial top to bottom bonds for tetragonal $BaTiO_3$. a) Impurity-atom contribution with the impurity atom, 0 carriers/u.c. and fixed lattice constants (white circles), b) Charge-carrier contribution with the impurity atom, 0.125 carriers/u.c. and fixed lattice constants (grey circles) and c) Lattice-constant contribution with the impurity atom, 0.125 carriers/u.c. and free lattice constants (black circles). The impurity atom is indicated on the x-axis. Each system corresponds to a 2×2×2 supercell and therefore has eight bond ratio values (for its eight transition metals), partly overlapping. Pure $BaTiO_3$ in tetragonal and pseudocubic symmetry (abbreviated *tet* and *cub*) as well as background-charge doped tetragonal systems (holes: h^+, electrons: e^-) are given as a reference.

Density of States

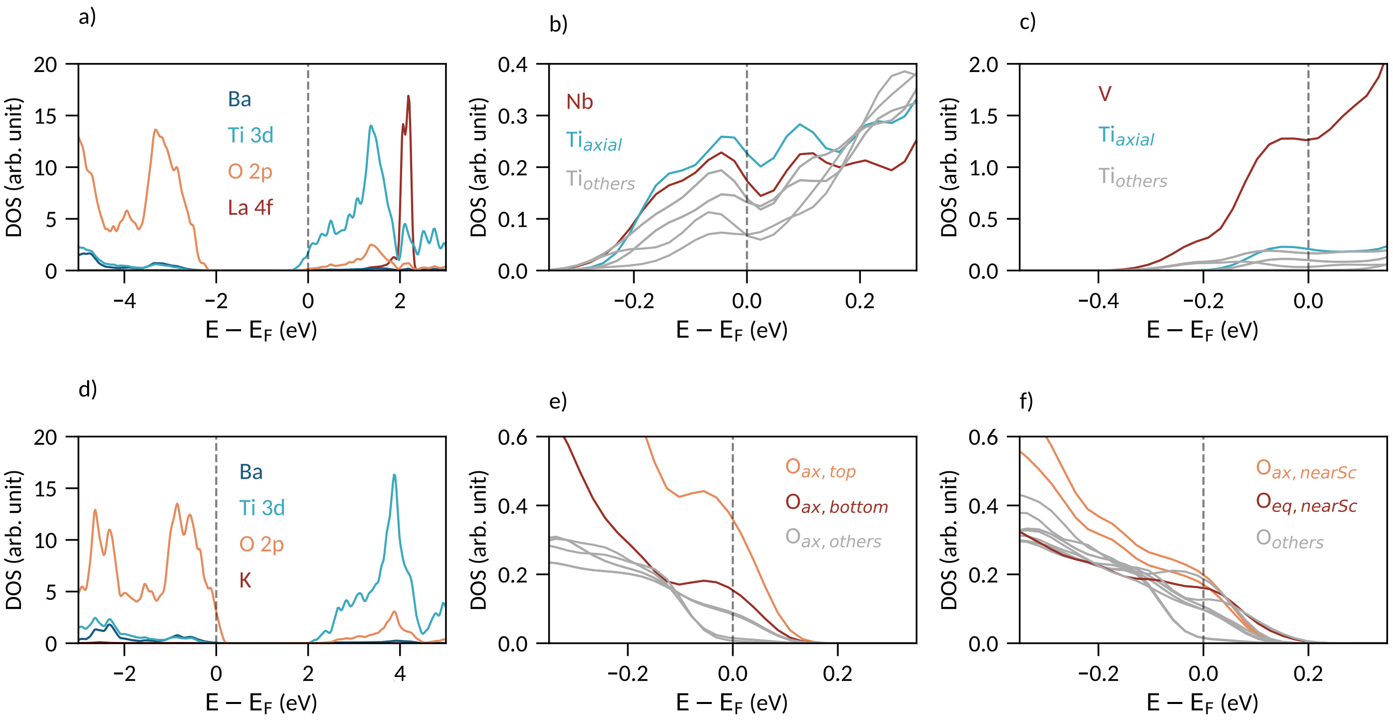

Figure 6: a) Density of states for La-doped $BaTiO_3$ (with one La in a 2×2×2 $BaTiO_3$ supercell with fixed lattice constants). As the La introduces one valence electron into the system, the Fermi energy lies in the conduction band. The La f-states lie at higher energies and the main contribution to the conduction band comes from the Ti d orbitals. b) Transition-metal site-resolved density of states of Nb-doped $BaTiO_3$ (BTNO with one Nb in a 2×2×2 $BaTiO_3$ supercell and fixed lattice constants). The region around the Fermi energy in the conduction band is shown. Two sites, the Nb and its axial neighboring Ti along the polar axis, have particularly large contributions. c) Transition-metal site-resolved density of states for V-doped $BaTiO_3$ (with one V in a 2×2×2 $BaTiO_3$ supercell with fixed lattice constants). The conduction band region around the Fermi energy is shown. The V has a particularly large contribution, whereas the its axial neighboring Ti along the polar axis hardly contributes to the conduction band. d) Density of states for K-doped $BaTiO_3$ (BKTO with one K in a 2×2×2 $BaTiO_3$ supercell and fixed lattice constants). One valence electron is depleted from the system so that the Fermi energy lies in the valence band. The K does not affect the DOS around the Fermi energy. e) Axial oxygen site-resolved density of states of Al-doped $BaTiO_3$ (with one Al in a 2×2×2 $BaTiO_3$ supercell with fixed lattice constants). The most contributing sites are the axial oxygens around the Al atom (top and bottom). f) Oxygen site-resolved density of states of Sc-doped $BaTiO_3$ (BTSO with one Sc in a 2×2×2 $BaTiO_3$ supercell and fixed lattice constants). The most contributing sites are the axial and equatorial oxygens around the Sc atom. All the supercells have tetragonal symmetry. All DOS are computed with a 12×12×12 k-point grid.
